# Successful Aging and Chronic Osteoarthritis

**DOI:** 10.3390/medicines5030105

**Published:** 2018-09-19

**Authors:** Ray Marks

**Affiliations:** 1 Department of Health and Behavior Studies, Columbia University, Teachers College, New York, NY 10027, USA; rm226@columbia.edu; Tel.: +1-212-678-3445; Fax: +1-212-678-8259; 2 Department of Health, Physical Education & Gerontological Studies and Services, City University of New York, York College, New York, NY 11451, USA

**Keywords:** aging, disability, healthy aging, osteoarthritis, pain, rehabilitation, successful aging

## Abstract

**Background**: Aging is commonly accepted as a time period of declining heath in most cases. This review aimed to examine the research base concerning the use of the term ‘successful aging’, a process and outcome deemed desirable, but challenging to attain. A second was to provide related information to demonstrate how health professionals as well as individuals can aim for a ‘successful aging’ process and outcome, despite the presence of disabling osteoarthritis. **Methods**: Information specifically focusing on ‘successful aging’ and the concept of improving opportunities for advancing ‘successful aging’ despite osteoarthritis was sought. **Results**: Among the many articles on ‘successful aging’, several authors highlight the need to include, a broader array of older adults into the conceptual framework. Moreover, conditions such as osteoarthritis should not necessarily preclude the individual from attaining a personally valued successful aging outcome. **Conclusions**: Pursuing more inclusive research and research designs, and not neglecting to include people with chronic osteoarthritis can potentially heighten the life quality of all aging individuals, while reducing pain and depression, among other adverse aging and disability correlates among those with osteoarthritis.

## 1. Introduction

In the 16th century, it was reported that the explorer Ponce de Leon marched in search of the fountain of youth, only to discover death. Intruding into Indian territory located in Florida, he was killed by an arrow at the age of 47.

However, due to declines in infant mortality rates and better health care in general, the population of older Americans, as well as those in most other nations, is rapidly increasing, even though no fountain of youth has been uncovered. Indeed, the longer life sought by de Leon is being experienced by millions of older persons in the United States (US) and worldwide. In this regard, current data in the US show many older adults not only living into their second 50 years, but that some have entered their ‘third’ 50-year life stage. By 2050, the US census bureau predicts the number of centenarions in this country alone could exceed four million [1], even if longevity is clearly being impacted negatively by obesity, violence, and opioid epidemics, among others.

While the aging process seems inevitable though regardless of longevity improvements, the question arises as to whether this programmed phase of growth to maturity, must inevitably lead at some point to a functional decline. This present overview focuses on the concept of ‘successful aging’ as an idea that can be attained, if one can potentially impact or slow this genetically programmed process of death and debility, and if this is indeed a desirable process. It explores what the term means precisely, if this can be achieved readily, and how clinicians may help aging adults to adapt to age-associated changes and to achieve less severe or fewer age-associated deficits or levels of disability than would otherwise occur without intervention, in general, and in the context of the most prevalent joint disease among older people, known as osteoarthritis. Since the concept of optimum health for all is an acceptable current public health goal, the idea that people with health conditions can still age ‘successfully’ must be considered. Moreover, if the term only applies to those who are disease free, very few elders will qualify, and it will be hard to explain how some disabled elders would classify their lives as successful or highly successful, regardless of age, which is not uncommon, and vice versa. In this respect, the key behaviors individual’s themselves can participate in or avoid in order to obtain a more successful, rather than a less ‘successful aging’ process and outcome, regardless of health status, such as the participation in appropriate levels of daily physical activity, are also highlighted. Taken as a whole, this present overview, while clearly not a meta analysis, may still be of potential relevance to older adults, as well as their families, and caregivers, or anyone working or living among the increasing numbers of older adults, especially those who have osteoarthritis of one or more joints, and chronic pain, presently a leading public health problem of epidemic proportions, and one where almost no report on the topic exists.

## 2. Methods

### 2.1. Data Sources

Related understandings and research as well as relevant materials were sought and downloaded from the PUBMED and Academic Search Complete data bases using the key terms, successful aging, healthy aging, and osteoarthritis, alone or in combination. An array of relevant papers were flagged, and after reading these, only those that focused on the current topics of interest specifically were eligible. All forms of research were deemed acceptable however, and those articles deemed of high relevance were further scrutinized for salient facts, ideas, and implications for advancing ‘successful aging’.

### 2.2. Procedures

Articles retrieved were categorized according to themes detailing the key concepts and findings of interest. This research encompassed articles that focused on ‘successful aging’ in general, the nature of osteoarthritis pathology, and the treatment and prevention of this disorder in relation to ‘successful aging’. Only a narrative review of the diverse oftentimes confusing material was undertaken, and no other disease specific entity other than osteoarthritis was included, although obvious overlaps among comorbid chronic conditions prevail. However, this work strove to solely examine this most salient costly disease disabler among older adults, regardless of where these populations reside, and the nature of osteoarthritis, in the context of aging ‘successfully’ with the view of possibly enabling a more inclusive conversation and body of research on this topic, along with possible encouragement to many aging adults and their caregivers.

After reviewing the widely heterogeneous data, which basically defied summative analysis, it was decided to focus the discussion on identifying how the original key concepts of ‘successful aging’ could be extended to encompass aging adults with osteoarthritis to compensate proactively to minimize the impact of their pathology, and heighten their subjective perceptions of being successful ‘agers’, despite this disability. For a very thorough review of the concept of ‘successful aging’ and its many varied interpretations, the concept paper by Flood [2] and the review paper by Martin et al. [3] are strongly recommended.

## 3. Results

### 3.1. Overall Findings

The literature search and the material extracted from that search, while not necessarily representing all publications in the field, showed that in general, there are many research reports that encompass one or more aspects of the theme of ‘successful aging’, but there is no comprehensive universally accepted model of the concept, and the concept is defined by a myriad of differing definitions, such as having achieved successful adaptations to an aging body [4], the absence of disease and disability [5], resilience/adaptation [6], and selective optimization with compensation [7]. As well, an array of research approaches, such as in-depth interviews conducted face to face or by phone [4,5,8], focus groups [6]; questionnaires [7], life story interviews [9], concept analysis [2,10], literature reviews [3,11,12,13,14] prevail. In addition, most studies examined small convenience samples of healthy cohorts of varying ages, and very few reports could be found that were devoted to the application of the concept of ‘successful aging’ to the osteoarthritis population. Moreover, very few studies discussed or compared interventions across time to assess the value of one or more identified potentially modifiable ‘successful aging’ determinants in the process of either healthy aging or aging in selected disadvantaged populations. Another feature was that very few studies examined the attribute and meaning of ‘successful aging’ from a holistic perspective, or from a more positive inclusive balanced perspective, rather than solely from a negative perspective.

### 3.2. Selected Aging Perspectives

Although data reveal that approximately 80% of older adults or those 65 years of age or older have at least one chronic health condition, and about 50% have at least two or more of these conditions, as well as being at risk for infectious diseases and injuries such as falls, contrary to the stereotyped myth that aging is synonymous with an overall health decline, most adults are found to be healthy and can function at a high level. As well, research shows neither muscle function, nor memory loss is inevitable, and that physical and cognitive power does not need to decline over time, as previously believed.

Rather, while acknowledging relevant age-associated health changes do occur, positive aspects of aging, and how to prevent or deal with these inherent physiological changes, as well as any health problems that are incurred over time, provides an alternative perspective known as ‘successful aging’, which is seen as a process, as well as an active state of being. Indeed, although many myths and misconceptions about aging as a state of decline clearly prevail, this idea of ‘successful aging’ is not merely an academic one, because if one examines the leading causes of death among adults ages 65 and older, which are heart disease, cancer, stroke, chronic lower respiratory diseases, Alzheimer’s disease, diabetes, influenza and pneumonia, and unintentional injury, none of these health conditions are simply inevitable consequences of aging [15]. Indeed, while it is true most chronic illnesses, once diagnosed in older adults are generally incurable, and can worsen over time, the key causes of most of these chronic illnesses and their morbidity rates as demonstrated in most recent nationwide and global public health research sites and data, are lifestyles and behaviors, changeable environmental agents, modifiable factors such as poverty, and other social factors, rather than genetic factors-once attributed an even role in determining longevity (e.g., smoking behaviors, eating behaviors). Additionally, most underlying causes or risk factors are amenable to prevention, intervention, remediation, elimination, or palliation, even if they are genetic (e.g., mastectomy for those with certain breast cancer genes, trauma prevention in those predisposed to genetically determined forms of osteoarthritis). Richardson et al. [16] also note that it is common for people with chronic conditions to report their health as good, even though models of healthy aging do not account for this.

Consequently, although many examples of debilitated older adults can be found, data show the majority of the older population to be neither debilitated, nor disabled. They may clearly be in their eighties but remain actively employed, they may be serving at the community level in volunteer endeavors, or involved in creative tasks, politics, education and other endeavors. Other data show that much of the innovation, beauty, science, and laws that prevail today are due to the efforts of talented or motivated people, who pushed themselves and developed their potentials, even though they were already deemed to be ‘senior’ citizens.

It can further be shown that many older adults, not only exhibit a complete ‘lack’ of any identifiable or seriously disabling disease or exposure to trauma, but that they may be more fit and functional than those in their 30s as a result of adopting a sound lifestyle. Even though bodily systems do still change over time, additional research shows a steady rate of decline from age 30 to 90, rather than any steep or rapid decline. These changes may also not occur simultaneously in all body systems, and even if they do change and influence the onset or risk of disease and dysfunction, these changes may still be reversible, e.g., abnormal glucose levels can be impacted favorably in many cases of pre-diabetes.

On the contrary, since change is a constant in life, regardless of the stage of development or growth, a philosophy that enables the individual to adapt to any special challenges may yet foster a meaningful existence, rather than an inevitable and painful declining, and depressive existence, even though there is much more research on aging that deals with patients, and their hospitalization or experience in the nursing home than their longevity and health attributes. Unfortunately, gerontology, or the study of aging, largely focuses on the negative aspects of aging, while geriatrics is concerned with treating problems related to aging, rather than preventing these, as well as on disability or senescence.

However, in 1984 John D and Catherine T. MacArthur gathered a group of scholars to develop a new set of insights regarding aging. Their mission was to stress the positive aspects of aging and to examine factors permitting certain individuals to function effectively, both physically and mentally in old age. More recently, Thompson et al. [17] found that even though polynomial regression of cross-sectional data obtained from 1546 individuals aged 21–100 years suggested a possible age-associated accelerated deterioration in physical and cognitive functioning, averaging 1.5–2 standard deviations over the adult lifespan, there appeared to be a linear improvement of about 1 standard deviation in various mental health attributes over the same life period.

### 3.3. Successful Aging

As reported in an initial publication in 1987 by the MacArthur group detailing the concept of ‘successful aging’ that followed their research efforts to better understand factors influencing positive health outcomes, despite aging, the idea put forth was that one did not have to age in a negative sense, but one could grow old, while maintaining their health, strength, and vitality, and could hence be deemed to be a successful ‘ager’ [18]. Since that time many hundreds if not thousands of articles related to this 1987 publication, and its dominant theme, along with its theoretical correlates have discussed this concept of ‘successful aging’, also known as healthy, active, productive, optimal, vital, or positive aging [3,19,20], among other definitions. This ever growing body of research continues to enlarge upon and expand on the early concept of ‘successful aging’, a viewpoint of aging built on the idea of harnessing the untapped resources of middle-aged adults and older individuals to enhance their well-being as they aged. It was a view encompassing attempts to maximize the potential of the aging adult to adapt to change, despite any physiological decline, and in so doing, to boost their chances of increasing their life satisfaction, mastery, growth, and longevity, as key components of ‘successful aging’, rather than succumbing to disablement and depression.

One overriding tenet of this idea was that aging in the negative sense was not inevitable, but was more likely than not to depend at least partly, on individual choices and behaviors, as well as effort. A second premise was that a broad focus of health issues, as well as more efficacious health policies, basic education that promotes health literacy, quality housing, medical care, and appropriate employment opportunities, where desirable, might substantially reduce impediments to a meaningful and high quality experience in later life, thus promoting the idea of ‘successful aging’ or aging well through proactive adaptation processes [3] as an achievable goal.

However, Cosco et al. [11] noted that even though almost half a century had elapsed since the inception of the term ‘successful aging’, no clear definition and hence no unified understanding of the underlying framework and concepts underpinning ‘successful aging’ could be readily identified. After conducting a systematic review using MedLine, PsycInfo, CINAHL, EMBASE, and ISI Web of Knowledge and a search that focused on quantitative operational definitions of ‘successful aging’, 105 such definitions, across 84 studies, using unique models were observed. A further analysis showed 92.4% or 97 studies included physiological constructs (e.g., physical functioning), 49.5% or 52, engagement constructs (e.g., involvement in voluntary work), 48.6% or 51 well-being constructs (e.g., life satisfaction), 25.7% or 27 personal resources (e.g., resilience), and 5.7% or 6 focused on extrinsic factors (e.g., finances). Thirty-four definitions consisted of a single construct, 28 of two constructs, 27 of three constructs, 13 of four constructs, and two of five constructs. The operational definitions utilized in the included studies identified between <1% and >90% of study participants as ‘successfully aging’. Based on these results, it appeared that the concept of ‘successful aging’ was clearly not a universal concept, and possibly one that was not always attainable, but its definition depended on what was being studied, who was being studied, or how the research was operationalized, among other factors. The idea that ‘successful aging’ is a multidimensional one, could consequently not be validated, and with no consistent research approach, no consensual definition of ‘successful aging’ or a framework for practice could be identified.

Earlier, Depp et al. [21] who similarly examined definitions and predictors of ‘successful aging’ using a large set of quantitative studies found 28 studies with 29 different definitions that met the researchers criteria. Even though most investigations used large samples of community-dwelling older adults, the mean reported proportion of successful agers was 35.8%, but this figure varied widely, and may have depended on the nature of the study, and the multiple components of ‘successful aging’ definitions identified. The most frequent favorable correlates of the various definitions of ‘successful aging’ were age (young-old), nonsmoking, and absence of disability, arthritis, and diabetes. Moderate support was also found for a positive role of greater physical activity, more social contacts, better self-rated health, an absence of depression and cognitive impairment, and fewer medical conditions in fostering ‘successful aging’. These aforementioned data results were mostly based on examining older adults without disability who still portrayed quite a low response to the extent of perceiving they had aged successfully.

However, regardless of definition and clarification of what constitutes ‘successful aging’, Tesch-Romer and Wahl [22] have argued that even if changing environmental settings, societal policies, and fostering individual life styles will significantly extend the number of healthy life years, recent epidemiological research confirms the dilemma that the ongoing extension of life expectancy prolongs not only the years in good health, but also those in poor health. This group thus argued that Rowe and Kahn’s ‘successful aging’ model 2 is still not able to cover the emerging linkage between increasing life expectation and aging with disability and care needs, and presents a clear limitation in this regard. Instead, this group suggested a set of propositions be forged towards a more comprehensive model of ‘successful aging’, and one that would capture desirable living situations including those that encompass aging adults with disabilities and specific care needs. They consequently went on to describe a number of individual, environmental, and care related strategies and resources for promoting autonomy and life quality in the face of disabilities and care needs in late life, putting emphasis on inter-individual differences and social inequalities, and that was designed to expand upon the traditional concept of successful aging, but to do this in light of current aging science and aging population needs.

Although Gopinath et al. [23] found physical activity helped to foster ‘successful aging’, this group who attempted to determine the features of ‘successful aging’ through interviewer-administered questionnaire, still classified this concept as the absence of: depressive symptoms, disability, cognitive impairment, respiratory symptoms and systemic conditions (e.g., cancer, coronary artery disease), thus excluding almost all aging persons from achieving this state.

Yet, other determinants of ‘successful aging’ that have emerged from the research may or may not be helpful. These include, the adoption of proactive lifestyles that enhance health, actions that correct for health-associated behavioral risks, making healthy choices the only choice, and participating in stimulating cognitive activities. Other theories imply that eliminating factors that increase the risk for disability, or in fact increase its rate of progress-if present-such as stress, substance abuse, and infections, while fostering high quality social support, community based programs, financial security, and in the case of the individual having them adopt a positive, rather than a negative self-concept [4] are likely to be very influential in efforts designed to foster more successful rather than less successful aging outcomes. The availability of appropriate information and other resources, as well as uplifting opportunities to engage with others, may similarly enable more ‘successful aging’ across the lifespan [4], regardless of health status, than would otherwise be experienced.

The concept of ‘successful aging’ itself, however, is currently ill defined and may mean different things to different persons, for example those from different cultures may not emphasize the same subjective or objectively defined attributes that are perceived as elements of ‘successful aging’ [4,5]. As well, a high degree of diversity in the perception of what this concept would mean personally, and whether this can be attained, if desirable may prevail, regardless of culture, health, and extrinsic factors. The studied attributes of ’successful aging’ are also highly heterogeneous and range from ‘having good health’, to ‘having a sense of purpose’, and autonomy, among others [4], such as the absence of disease and disability [5], and the ability to maintain physical and cognitive functioning [5]. Adding further confusion is research showing these attributes and others can readily fluctuate depending on age-wherein ‘successful’ agers tended to be younger and report more frequent engagement in lifestyle activities than older subjects [7], gender [5], living circumstances [9], and the extent of any disability and pain [9], among other factors.

In addition, another term related to the concept of ‘successful aging’, namely, ‘healthy aging’, similarly hosts a number of definitions and components of well-being that differ depending on the stance of the researchers, and their specialty. This latter concept also differs somewhat from that of ‘successful aging’, and is suggested to denote the ability of the individual to modify, reassess and redefine oneself-but does not preclude disease states. Resilience, adaptation and compensation are other important characteristics of healthy aging that appear valid, but again these may vary dependent on the values held by different societies, as well as societal and individual expectations, and the role of family, social engagement, among other factors. Another view is that if disease is seen as a normal part of aging-rather than modeling ‘successful aging’ on the basis of freedom from disease, Amin [4] implies it may be fruitless to identify whether any universal set of ‘successful aging’ constructs prevail. Rather, the individual’s perception and how they define ‘successful aging’ or ‘aging well’ must be uncovered through careful interactions and communication efforts.

In short, the concept of ‘successful’ or healthy aging, or that of a ‘good old age’ is depicted as multidimensional. Its scope and meaning may depend however on what premises researchers decide to test, the type of research questions posed, the sample numbers and characteristics. Results may also depend on the individual and her perceptions, as well as how practitioners and researchers view this concept [3]. However, despite a myriad of views, and discrepancies among the diverse biomedical, psychological, and lay perspectives [21], it appears that to promote ‘successful aging’, something more than personal attributes and understandings can determine this life outcome. For example, an empathetic provider-patient relationship and access to quality care and living conditions and taking cultural preferences and views into account can help even those who are disabled to be more successful than not. As well, a focus on the socio-emotional aspects of health, as well as the physical aspects of health will clearly be more helpful than not, regardless of level of ability or disability.

Elements of ‘successful aging’ may also include sound nutrition practices, an active lifestyle, having a degree of financial security, and sound sleep hygiene strategies, and in our view are just as relevant to those with health conditions as those who have no health disability. Efforts to reduce or minimize anxiety, depression, and low self-concept are additional strategies that can be applied, regardless of health status. According to findings by Richardson et al. [16] who examined how resilience relates to how people consider themselves to be well, regardless of their adverse health condition, the experience of adversity is influenced by context and meaning, findings which support a broader version of resilience that incorporates vulnerabilities, and should not undermine an older person’s sense of a resilient self.

Zanjari et al. [14] who examined 76 topical articles eligible for inclusion in an integrative review, found 14 subcategories and 5 main categories of ‘successful aging’ to prevail, including social well-being, psychological wellbeing, physical health, spirituality and transcendence, and the environment and economic security. Another study by Parslow et al. [24] found factors measuring mental, physical and social health can all contribute significantly and independently to ‘successful aging’ and that the presence of chronic health conditions does not necessarily preclude high levels of well-being in older individuals. Another view is that disease and disability might be viewed as a normal part of aging, and thus freedom from disease or longevity is not necessarily of high import to the same degree in all cultures [4].

In the next section, we argue that contrary to early beliefs that saw ‘successful aging’ as a state where the individual was disease free, attaining this state is in the eyes of the individual, and its attainment should not be neglected in the presence of an irreversible disease.

### 3.4. Osteoarthritis

Osteoarthritis is a painful disabling joint disease that predominantly affects more than half of those adults who are 65 years of age or older. An incurable disease that often affects more than one joint, the condition can be extremely debilitating and disabling is one where few risk free treatment strategies exist. Moreover, medications applied to alleviate disease symptoms are often contra-indicated or induce unwarranted side-effects, and surgery is not always effective or warranted. Given that many with this physical condition also show signs of depression [25], a diminished work capacity, restrictions of social activities [26], and most have one or more comorbid chronic diseases, and/or are overweight or obese, and all these components of the illness interact to often increase the severity of the condition [26], the idea of ‘successful aging’ is clearly one that is highly contrary when conceived as an ideal for those with this intractable condition.

So what can be done to assist the aging adult with osteoarthritis to maximize their overall well-being, while minimizing their disability in an effort to still attain a ‘successful aging’ state of being? Clearly, the disease, a multi-dimensional one that includes psychological as well as physical symptoms is not reversible, and can readily spread from one joint to another, while progressively degenerating processes proceed unhindered. To favorably alter the potential downward spiral, and ensure the goal of ‘successful aging’, or ‘healthy aging’, research shows this is feasible in light of what we do know, even though this requires very careful assessments, and interventions that are not only carefully construed, but are carefully tailored and titrated.

Fortunately, as with healthy adults, an extensive array of related research testifies to the enormous benefits of exercise adoption and muscle conditioning in this context, which is consistent with the idea that the health condition is more likely to occur in the presence of weak muscles or muscle pathology than not. As well, the finding that those who exercise, are more likely fewer weight issues, and are better off than those with high body weights speaks to an important role for both exercise and weight control in efforts to attain a successful health outcome, despite the disease. Other salient strategies related to joint protection efforts, such as the avoidance of undue joint stressors, vigorous exercises, and excess use of opioid narcotics, among other approaches will help foster the ability to pursue health goals more readily than not. As well, the use of heat and cold applications-as required, along with selected oral and topical medications, stress reduction efforts, ergonomic adaptations of the home and workplace, adaptive devices, and social support are found to help assist the patient with this condition to substantively raise their life quality, and to possible attenuate the disease symptoms, as well the risk of injury and comorbid health conditions such as diabetes. As with all attempts to foster ‘successful aging’, early intervention in childhood and adolescence is indicated, even though osteoarthritis is often considered an age-associated disease.

In sum, disabling and debilitating osteoarthritis, the most common joint disease affecting older populations, may not be inevitable, in all cases, and even when present, its severity and extent can be attenuated and may be amenable to amelioration if careful evaluations followed by carefully construed and timely treatment strategies are forthcoming and sustained where necessary. Some of the non-operative approaches for helping the osteoarthritis sufferer to maximize their health outcomes, may yet promote feelings of ‘success’ and control over their condition in the face of a very demoralizing disease state.

It is also reasonable to suggest that providers who adopt realistic outcome goals for effective rheumatic pain management for their patients, and help them to moderate any erroneous constraint beliefs [25], and to convey this clearly to the patient will help them to avoid unrealistic expectations, as well as excess pessimism, for example, believing joint surgery will be restorative or painfree, or that opioids that relieve pain are safe, or believing their condition is not amenable to intervention. In addition, even though pain—the symptom of most concern to patients is unlikely to be completely abolished, regardless of intervention, helping patients to understand the key determinants of pain, and what to avoid, or act on is very key to success. As well, to foster adherence behaviors, barriers to effective pain management from both the patient and the healthcare professional perspectives should be minimized. In this respect, acceptance therapy, where the patient is encouraged to accept their situation, but not to lose sight of their goals, appears very worthy of consideration and application. Joining a support group or acting as an advocate for others or journaling have been shown to advance well-being despite the presence of disability, and regardless of age.

It is also reasonable to suggest that providers who adopt realistic outcome goals for effective rheumatic pain management for their patients, and help them to moderate any erroneous constraint beliefs [25], and to convey this clearly to the patient will help them to avoid unrealistic expectations, as well as excess pessimism, for example, believing joint surgery will be restorative or painfree, or that opioids that relieve pain are safe, or believing their condition is not amenable to intervention. In addition, even though pain-the symptom of most concern to patients is unlikely to be completely abolished, regardless of intervention, helping patients to understand the key determinants of pain, and what to avoid, or act on is very key to success. As well, to foster adherence behaviors, barriers to effective pain management from both the patient and the healthcare professional perspectives should be minimized. In this respect, acceptance therapy, where the patient is encouraged to accept their situation, but not to lose sight of their goals, appears very worthy of consideration and application. Joining a support group or acting as an advocate for others or journaling have been shown to advance well-being despite the presence of disability, and regardless of age.

**Figure 1:**
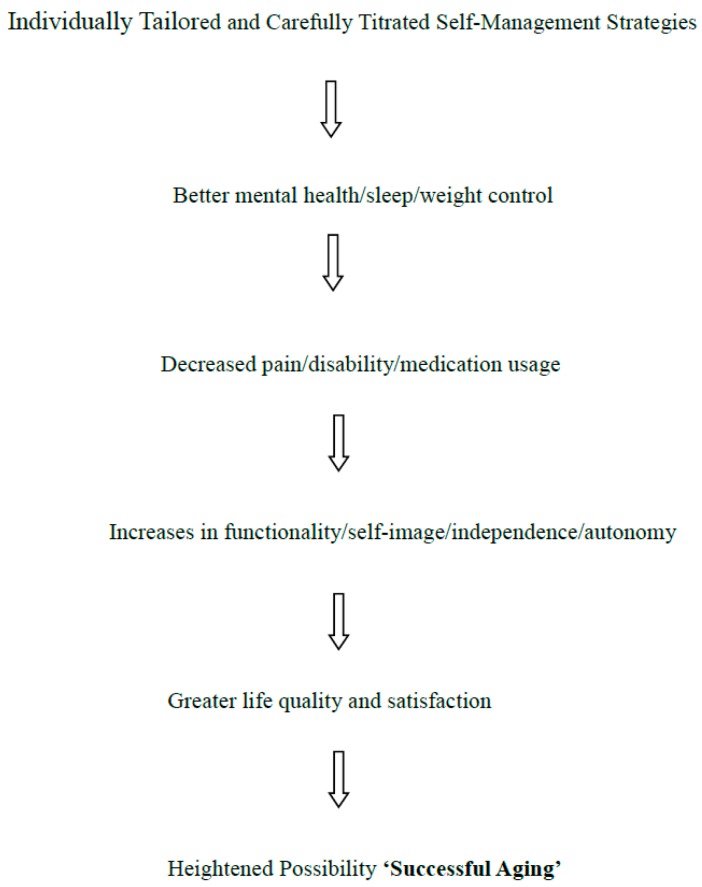
Conceptual model of anticipated outcomes of carefully tailored and titrated management.

In short, as per Figure 1 shown below, it is highly possible for an older adult with osteoarthritis, to age ‘successfully’ in our view, especially if they try to proactively contribute to their own wellbeing on a consistent basis, in conjunction with their provider’s recommendations, despite an associated array of cognitive and physical challenges. This ability to manage their health condition is not a given though, and may depend not only on the degree of presiding impairment, but on other intrinsic factors such as health beliefs, motivation and ability to comply with their providers’ recommendations, and extrinsically on the degree to which providers are knowledgeable and accessible, program attributes are tailored and clearly imparted, and the degree of societal or family support. More direct interventions include the use of supplements, topical gels, maintaining a healthy weight and blood pressure, plus home improvements and worksite adaptations to minimize joint and overall stress. In the meantime, an acceptance of a broader definition of ‘successful aging’ is fundamental in this regard in this author’s view, along with the understanding that the disease affects the whole body, not just a single joint, including the neurological, structural, and cardiovascular components of the body, and their relationship to physical, mental, emotional, and social wellbeing, and that it is not inevitable or subject solely to deterioration.

## 4. Discussion

By 2030, the numbers of older adults in the US will more than double to about 71 million [1]. However, not all will age successfully, even if they have no distinctive co-morbid health condition. On the other hand, this rapidly increasing number of older Americans, as well as those in other areas of the globe, will probably have high rates of chronic diseases, which may be barriers to the achievement of a fully functional life, unless a broader vision of ‘successful aging’ is considered, along with heightened early preventive health promotion efforts in this author’s view.

Since this aging nation’s efforts to ensure the achievement of ‘successful aging’ for all have clearly yielded suboptimal results, along with associated potentially modifiable economic and social costs, a deeper consideration of the ‘successful aging’ theme and opportunities in the research, academic spheres, psychology and policy arenas to advance this concept appears imperative. However, though initially deemed an essential component of ‘successful aging’, namely, keeping older adults healthy and disease free, this outcome, while clearly desirable, is generally not readily observed at all today, despite modern medicine advances, and positive longevity gains, overall. Indeed, a pure aging process that has not been influenced by illness, trauma, or genetic determinants, is clearly very unlikely to prevail for most, especially if an older person has developed one or more chronic health challenges, such as osteoarthritis in their early teens or adult years.

Yet, if the goal of ‘successful aging’ is to enable optimal states of well-being across the life span including the later stages of the life trajectory, the perception that all individuals can ‘age’ optimally, may permit a more inclusive definition of this concept that embraces the potential of all citizens and does not discriminate between the presence or absence of prevailing health problems and is very likely to be more advantageous than not in this author’s view. To this end, this review has described some aspects of ‘successful aging’ also termed ‘healthy aging’ in an effort to garner some insight into fostering this concept for people with chronic osteoarthritis. Based on an in depth review of all available literature, it is clear no finite definition or accepted set of constructs prevails in this regard, despite nearly 30 years of research. There is almost no literature on this concept that speaks to osteoarthritis sufferers even though more than half of aging adults will have this condition.

Since the attributes of resilience, autonomy, acceptance, the ability to cope to achieve personally valued goals, and independence are valued attributes of ‘successful aging’ [4,6,10] these traits alone, among others, such as perceived self-efficacy for coping with life in general, which can be strengthened, can clearly pertain to all aging adults, regardless of physical health status, and their attainment might potentially yield more success than not across all stages of the older adults’ life trajectory. On the other hand, more health education, and availability of health resources and services, rather than seeking genetic solutions, or magic anti-aging bullets, would go a long way to helping both those adults who are unimpaired as well as those who are impaired to age gracefully-or successfully. Placing emphasis on what success means to the individual at the outset though-can surely help practitioners to guide citizens to achieve personal values that would denote success to them, rather than attempting to follow prescriptive or preconceived models. In the case of the osteoarthritis sufferer, one who proactively tries to maintain a full and independent life by means of their own efforts to accommodate certain physical changes, to prevent harm, and to promote regenerative or reparative states, the individual may feel very satisfied and thus successful-all things considered as outlined in Figure 1. On the other hand, even if their osteoarthritis condition is manageable, but is viewed in a negative way by the society, social, political, and medical systems, their level of depression might be heightened-and their self-worth lowered with dire ‘aging’ consequences. As well, if nothing is done to translate and apply evidence supporting primary prevention strategies, implemented early on in life, and an ecological viewpoint of health outcomes and determinants that fosters optimal safe and enriching living environments, offsetting the prevalence as well as the severity of this condition through self-care activities alone seems unlikely. These strategies may include, but are not limited to the avoidance of injury, drinking and driving, along with better public health protection laws, fighting against criminal victimization of older people, avoidance of over- or under-eating, and sedentary activities, improved undergraduate and graduate education about aging, and community-based health promotion programs. More marketing by Aging and Arthritis Foundations of what can be done to protect against osteoarthritis, and the importance of seeking help earlier rather than later is also recommended. As well, refuting the myth that nothing can be done for their health condition, that aging is inevitable declining process and others must be actively countered with science based data. As well, more efforts by physicians and rheumatologists on efforts to combat or minimize depression and anxiety and improve coping skills in this group, as in healthy elders, will undoubtedly be very helpful in advancing their perception of ‘success’ across the lifespan of their later years.

## 5. Conclusions

In the author’s opinion, ample research revealing the idea that aging is an inevitably declining state does not appear to be a valid one. Rather, when cases of individuals who do not appear to ‘decline’ objectively to any degree over time are examined, certain commonalities related to life style and behaviors, as well as socioeconomic opportunities appear more relevant than genetics. As well, equal numbers of those who are centenarians are found to have one or more health conditions, while other have few detectable problems. Not all centenarians however, are satisfied with their attainment, even if longevity is desirable, while others clearly take action overtly to prevent longevity. Thus, the idea that there is a one size fits all-or single unified formula for achieving ‘successful aging’ clearly seems elusive at present, as borne out by the research. However, even though no consensus on what should be studied in this realm is evident, and the varied samples and range of attributes studied to date may account for the lack of uniformity in this sphere, the idea that ‘successful aging’ is closely related to autonomy and goals such as physical and psychological functioning [21], suggests that this goal can be sought by most aging adults-or in fact promoted by states or countries-regardless of health status. Yet, very few related studies have actually examined aging individuals deemed to be impaired, and that take into account the fact that the mainstream cultural values of autonomy, and success in the context of aging are not likely to be uniform. The process of trying to examine only a selected number of possible ‘successful aging’ related determinants such as financial status, physical activity, body mass index, depression, participation in social activities with friends and family, number of yearly excursions, number of cardiovascular disease risk factors and adherence to the Mediterranean diet, may further limit understandings, and explain why these data when combined only yielded an index of ‘successful aging’ showing a 1/10-unit increase in the index was associated with 0.8 less annual visits to healthcare centers over 6 years (95% CI −1.3 to −0.2). [27]. Indeed, as outlined by Kim [28], as well as Brown and Bond [8], the findings suggest that the increased longevity of centenarians and adults over the age of 65, is affected by multiple social factors, such as support services for the elderly, and social well-being programs and policies, as well as the definition of ‘successful aging’ applied, and these factors warrant further study.

However, based on what we do know, it appears that all aging adults can improve their health outcomes through a variety of self-directed proactive strategies if they are mentally able, as well as motivated towards achieving the promised outcomes. Moreover, despite their challenges, older adults with osteoarthritis who consistently practice positive healthy behaviors, and have access to and can take advantage of clinical and community-based services are quite likely to be able to engage in and pursue a personally meaningful life, and to achieve an acceptable level of independence, if desired, and one they personally will deem to be ‘successful’, despite their challenges.

Yet, despite much research, the literature in support of this concept of ‘successful aging’ not only shows no consensus on its attributes, and few studies that considered ‘successful aging’ from a holistic perspective or that examined actual ability to function physically, mentally, economically, socially, and emotionally, either in the case of those deemed physically healthy or impaired. Instead, several researchers explored the dimensions of ‘successful aging’ through the lens of a small array of pre-determined themes, and even if personal themes of success were identified, very little work follow up work was done to test or validate these perspectives and what these denote in persons with disabilities, as well as what the concept does not denote, or why only very few older individuals who did not need home care services in the past 59 days were not categorized as successful agers [29]. As well, although having a spouse, good mental health status, and life satisfaction were assessed and found to be salient predictors of successful aging, the fact that only disease free individuals were examined precludes this finding from being generalizable. This series of prevailing ‘successful aging’ research approaches in general, has also been criticized by Thompson and Forbes [17] who felt ‘disease free’ older persons do not represent intrinsic or true aging attributes. As well, Stowe et al. [13] who examined ‘successful aging’ through a life-course lens urged caution in using the model in its current formulation owing to its emphasis on personal control over one’s later-life outcomes, and neglect of historical and cultural context, social relationships, and structural forces in influencing later-life functioning. Additionally, very few studies have examined the importance or role of educational attainment, health literacy, motivation for successful aging, the housing environment, food quality and availability, the possible influence of nutrigenomics [30], role loss, dependence, loneliness, medication related issues, comorbid and sleep issues in the context of moderating or mediating ‘successful aging’ in different age groups and genders [31]. Many if not all these factors may thus represent overlooked explanatory factors or ‘successful aging’ antecedents, as well as intervention points of specific import in efforts to optimize the health quality and independence of older people across the lifespan. Others points of consideration are highlighted in Table 1 below.

## 6. Future Research and Practical Challenges

Although this report itself is limited, and does not attempt any meta-analytic consolidation of the topic, it seems that much more effort to tease out and examine the concept of ‘successful aging’ alongside various aging, biological, psychological and sociological theories, technological influences, living circumstances, socially validated work opportunities, health care access, and culture among other extrinsic factors is needed to arrive at some meaningful individual, and if possible, national strategy or orientation towards fostering ‘successful aging’ for all as suggested by Martin et al. [3] and Martinson et al. [40]. To better define ‘successful aging’, more large scale interdisciplinary efforts, more study of young healthy and impaired adolescents and adults across the range of the aging continuum [12] in well-designed prospective and intervention studies that take lay views into account [31], along with efforts to acquire a better understanding the role of stress, depression, spirituality, the epigenetic and the social determinants of health are likely to prove helpful. As well, studying those with and without specific health issues including, but not limited to osteoarthritis, using an appropriate combination of biochemical, cognitive, and functional outcome variables to define ‘successful aging’ among diverse samples can possibly help to indicate potential areas for maximizing successful long term health outcomes in the future. More efforts too to discern which predictors of ‘successful aging’ are of greatest import, such as socioeconomic, socioenvironmental, educational factors, chronic exercise, nutritional, genetic, and health status factors, among others will undoubtedly be of additional help to both policy makers and clinicians in any future effort to advance this field beyond the theoretical level. The role of smart technology, stem cell research, regenerative medicine, inflammation control, nutrients and behaviors, early life health education, and robotics in expediting ‘successful aging’, mostly uncharted topic areas, but increasingly relevant, also warrants attention. However, even if all this is forthcoming, moving mainstream medicine to conceptually foster the idea of ‘successful aging’ for all across the lifespan, as an active endeavor, regardless of age, genetics, disease, injury, functional or cognitive status, is potentially the most critically needed focal point for advancing health and optimally successful longevity in all populations.

Additional challenges that might be considered in deriving an agreed upon understanding of ‘successful aging’ and one that can be examined systematically are:

▪ The failure to view the concept holistically across varying age and cultural groups, marital status, occupational prestige, years of formal education, race, annual income, and a variety of specific satisfaction with life measures related to successful aging, as well as spirituality [41,42].
▪ The diverse methods and measures used to examine ‘successful aging’.
▪ The widespread use of surveys to derive insights about ’successful aging’ rather than functional measures.
▪ Subjective and objective criteria of ‘successful aging’ may differ [43].
▪ Availability, accessibility, role of appropriate services, and needs are poorly explored correlates.
▪ Negative stereotypes and beliefs, control beliefs, about aging are unexplored factors.
▪ The commonly used operational definition of successful aging (high cognitive and physical function, low probability of disease, and active engagement with life) does not necessarily reflect the values of other cultures or those of older persons in the US [16].
▪ Model was primarily designed for high-income populations and may not be transferable to populations in low- and middle-income countries [44].
▪ Model has not considered role of environmental contaminants or hazards [44].

## Figures and Tables

**Table 1:** Selected successful aging studies and conclusions.

Researchers	Key Conclusions
Bowling et al. [32]	A model of successful aging needs to be multi-dimensional, incorporate lay perspectives, and use a continuum for success.
Hamid et al. [33]	Showed age, educational attainment, household income, and ethnicity were significantly associated with successful aging.
Iwamasa et al. [34]	Participants perceived successful aging as optimal functioning in: physical and psychological health, cognitive functioning, socialization, spirituality, and financial security. The content of each dimension represents both culture-specific and culturally-universal elements.
Parslow et al. [24]	Chronic illness is not necessarily a barrier to successful aging.
Katz et al. [35]	Inattention to intersecting issues of social inequality, health disparities, and age relations, and possible role of social exclusion in successful aging processes remain.
Lowry et al. [12]	Successful aging is a multidimensional construct that could be viewed as a continuum of achievement. Based on the disability model proposed by the WHO International Classification of Functioning, Disability and Health, successful aging includes not only the presence or absence of disease, but also aspects of mobility and social participation.
Ng et al. [36]	Although aging well socially (engagement with life) is as important as aging well personally (illness avoidance and functioning) (Rowe & Kahn, 1998) Results supported the differentiation of Rowe and Kahn’s engagement with life component into caring and productive engagements.
Jeste et al. [37]	Resilience and depression had significant associations with self-rated successful aging, with effects comparable in size to that for physical health.
Pietrzak et al. [38]	Interventions and policy initiatives designed to mitigate physical health difficulties and psychological distress and to enhance protective psychosocial characteristics such as resilience, gratitude, and purpose in life may help promote successful aging in these populations.
Vahia et al. [39]	Self-rated successful aging emerged as the primary downstream factor and exhibited significant partial correlations with psychosocial protective factors, physical/general status and mental/emotional status but not with cognitive ability.
